# Distinct and Overlapping Patterns of Acute Ethanol-Induced C-Fos Activation in Two Inbred Replicate Lines of Mice Selected for Drinking to High Blood Ethanol Concentrations

**DOI:** 10.3390/brainsci10120988

**Published:** 2020-12-15

**Authors:** Stacey L. Robinson, Ana Paula S. Dornellas, Nathan W. Burnham, Christa A. Houck, Kendall L. Luhn, Sophie C. Bendrath, Michel A. Companion, Honoreé W. Brewton, Rhiannon D. Thomas, Montserrat Navarro, Todd E. Thiele

**Affiliations:** 1 Department of Psychology & Neuroscience, The University of North Carolina, Chapel Hill, NC 27599, USA; slrobin@email.unc.edu (S.L.R.); lopera@email.unc.edu (A.P.S.D.); chouck@email.unc.edu (C.A.H.); kluhn@email.unc.edu (K.L.L.); sophiecb@ad.unc.edu (S.C.B.); macompan@live.unc.edu (M.A.C.); honoreeb@live.unc.edu (H.W.B.); thomasrd@unc.edu (R.D.T.); mthiele@email.unc.edu (M.N.); 2 Bowles Center for Alcohol Studies, The University of North Carolina, Chapel Hill, NC 27599, USA; 3 Department of Chemistry, North Carolina State University, Raleigh, NC 27695, USA; nwburnha@ncsu.edu

**Keywords:** HDID, c-fos, mice, ethanol, alcohol, drinking in the dark, taste aversion

## Abstract

The inbred high drinking in the dark (iHDID1 and iHDID2) strains are two replicate lines bred from the parent HS/Npt (HS) line for achieving binge levels of blood ethanol concentration (≥80 mg/dL BEC) in a four-hour period. In this work, we sought to evaluate differences in baseline and ethanol-induced c-Fos activation between the HS, iHDID1, and iHDID2 genetic lines in brain regions known to process the aversive properties of ethanol. Methods: Male and female HS, iHDID1, and iHDID2 mice underwent an IP saline 2 3 g/kg ethanol injection. Brain sections were then stained for c-Fos expression in the basolateral/central amygdala (BLA/CeA), bed nucleus of the stria terminals (BNST), A2, locus coeruleus (LC), parabrachial nucleus (PBN), lateral/medial habenula (LHb/MHb), paraventricular nucleus of the thalamus (PVT), periaqueductal gray (PAG), Edinger–Westphal nuclei (EW), and rostromedial tegmental nucleus (RMTg). Results: The iHDID1 and iHDID2 lines showed similar and distinct patterns of regional c-Fos; however, in no region did the two both significantly differ from the HS line together. Conclusions: Our findings lend further support to the hypothesis the iHDID1 and the iHDID2 lines arrive at a similar behavior phenotype through divergent genetic mechanisms.

## 1. Introduction

The predisposition to consume high amounts of ethanol in a short period of time, or ‘binge-like’ behavior, has been extensively demonstrated to serve as a predictive measure for the development of ethanol dependence and is further a dangerous behavior associated with numerous health risks [1,2]. While extensive work has demonstrated a genetic contribution to ethanol preference and predisposition to withdrawal from chronic ethanol use, factors underlying the drive to binge consume ethanol have received relatively less attention. To enable investigation into the genetic underpinnings of binge ethanol intake, the Crabbe research group developed the high drinking in the dark (HDID) strain, a line selected from an original heterogeneous breeding stock (HS/Npt or HS), for high blood ethanol concentration at the end of a four-hour binge period [3]. Following the creation of the HDID1 strain, a replicate breeding process was performed to create the HDID2 strain. To stabilize each genotype, the group then established an inbred version of each line (iHDID1 and iHDID2) which we utilize in this work [4]. While no rodent line can fully model the full spectrum of human alcohol use disorders, these lines present an opportunity to gain insights into genetically determined mechanisms which predispose individuals to achieve high blood ethanol concentrations in a short period of time.

Though generated using identical criteria, these two strains display significant differences in ethanol drinking pattern (microstructure) and brain gene coexpression networks relative to both the HS line and each other, suggesting polygenetic origins of the same behavioral end point [5,6]. The HDID1 line consumes ethanol in the same number of bouts as HS, but ingests larger amounts of ethanol during each individual bout [5]. This difference in bout size was not observed in water or saccharine consumption in this study, suggesting that this behavior is ethanol specific. This is notable as early ‘gulping’ of ethanol, or consumption of large amounts of ethanol in a single drinking bout, has been shown in non-human primates to be predictive of future chronic heavy drinking [7]. Further, larger bout sizes are associated with a relative increased ‘liking’ of ethanol which is thought to be driven by an increased experience of the positive or decreased experience of the aversive properties of ethanol [8,9]. This may suggest a pathway through which HDID1 animals are susceptible to heavy drinking, distinct from the higher number of bouts consumed by HDID2 animals [5]. Several studies have suggested that the HDID1 and iHDID1 lines may possess an intriguing resistance to the aversive properties of ethanol consumption. While HDID1 and iHDID1 mice develop a conditioned taste aversion (CTA) similar to that of HS mice following lithium chloride exposure, they display an attenuated CTA to 2 g/kg (but not 4 g/kg) ethanol injection [4,10]. This may suggest that relatively higher levels of ethanol intoxication are required to generate the experience of the aversive properties of ethanol in this strain. That no difference between the HS and the original HDID1 and HDID2 genotypes in ethanol conditioned place preference (CPP) were observed in this work further suggests a reduction in the aversive effects of ethanol, rather than an enhancement of the reinforcing properties, may underlie increased consumption. A lack of change in the relative level of anxiolysis induced by ethanol consumption or injection compared to HS mice lends further support to this hypothesis [11].

The more recently generated HDID2 line appears to display less ethanol-specific alteration in behaviors. HDID2 animals tend to consume high levels of ethanol through a larger number of similarly sized bouts relative to HS or HDID1 animals for both ethanol and water, suggesting that they may show a generalized increase in intake [5]. They further display a more generalized reduction in CTA behavior, showing less CTA to both 2 g/kg ethanol and lithium chloride exposure [10,12]. Similar to the iHDID1 line the iHDID2 strain possess a similar level of ethanol CPP and anxiolysis following ethanol exposure as HS animals, suggesting that this line may also have little relative change in processing the reinforcing effects of ethanol versus the parent HS line. Additionally, the iHDID2 line shows reduced sensitivity to the aversive effects of ethanol relative to the HS mice [4,11].

In the present study, we assessed acute ethanol-induced neural activation, as measure by c-Fos expression, in the HS, iHDID1 and iHDID2 lines. The guiding hypotheses of this study were that a) the iHDID lines would show significant differences in c-Fos activation relative to the HS line in brain regions modulating ethanol consumption (specifically those involved in the aversive properties of ethanol) leading to binge-like BECs and b), that the iHDID1 and iHDID2 lines would show significant differences in c-Fos activation in a subset of brain regions, which may provide insight into line differences in drinking microstructure and genetic networks outlined above. Given the attention dedicated in previous studies to the relative processing of the aversive properties of ethanol, this current work focused on c-Fos activation within multiple (though in no way inclusive of all) mid- and hindbrain regions associated with these properties. These doses were chosen based on previous work that assessed CTA sensitivity in the HS, iHDID1, and iHDID2 lines [4] The present results provide insight into the potentially divergent patterns of neural activity which underlie the same ultimate behavioral phenotype of binge-like drinking behavior in these two independently generated high drinking strains.

## 2. Methods

### 2.1. Animals

Inbred high drinking in the dark (iHDID1 (generation S26.F20), iHDID2 (generation S32.F10) and the founder HS/NPT (HS) mice (generation G95) were group housed 2–5 per cage from weaning until the beginning of experiments, with a room temperature maintained at 22 °C and a 12:12 h forward light/dark cycle with lights on at 7:00 am. Ad libitum Prolab^®^ RMH 3000 (Purina labDiet^®^; St. Louis, MO, USA) chow and water were available. All protocols were conducted under National Institute of Health guidelines and were approved by the University of North Carolina Institutional Animal Care and Use Committee (protocols 20-165.0 and 18-096.0).

### 2.2. Immunohistochemistry

A total of 15 male and 15 female mice (6–8 weeks old) from each independent lines (iHDID1, iHDID2) and HS/NPT (HS) were used in each dose condition for a total of 90 mice. Due to tissue processing complications, not all brains were viable for analysis for every brain area. Individual Ns for each genotype, treatment, and sex are provided in the results for each region.

One week before the experiments, mice were habituated to single housing. On the test day, mice were intraperitoneally (IP) injected with ethanol (Decon Laboratories, code 2801, King of Prussia, PA, USA) at 2 or 3 g/kg (made up as 20% (*v/v*)) or saline (Hospira, code NDC 0409-4888-02, Lake Forest, IL, USA) (equivalent to the 3 g/kg volume of liquid) two hours before perfusions. Mice were weighed and anesthetized with ketamine/xylazine (Ketaset, code EA2489-564, Kalamazoo, MI, USA; AnaSed, code NDC 59399-111-50, Lake Forest, IL, USA) overdose (100/10 mg/kg) and transcardially perfused with phosphate-buffered saline (PBS; Sodium phosphate monobasic, VWR Life Science, code 0571, Solon, OH, USA; Sodium phosphate dibasic, ACS, code 0404, Solon, OH, USA; Sodium chloride, Macron Fine Chemicals, code 7581-12, Radnor, PA, USA) for 1 min followed immediately by 10% formalin (Fisher Scientific, code SF100-20, Hampton, NH, USA) for 2 min using a Masterflex L/S perfusion pump (catalogue #7200-12, Cole-Parmer, Vernon Hills, IL, USA) in a rate of 7–8 mL/min.

Brains were collected and postfixed in 10% formalin for 24 h, and, next, were cryoprotected in 0.2 M Phosphate buffer (PB; Sodium phosphate monobasic, VWR Life Science, code 0571, Solon, OH, USA; Sodium phosphate dibasic, ACS, code 0404, Solon, OH, USA) at pH 7.4 containing 50% ethylene glycol (Fisher, catalogue #E178500, Hampton, NH, USA) and 0.01% polyvinyl-pyrrolidone (VWR Life Science, catalogue #EM-7220, Radnor, PA, USA). Brains were sectioned using a vibratome into 40 μm slices (model VT1000 S, Leica Biosystems, Buffalo Grove, IL, USA). Tissue was placed in cryopreserve and stored in a −20 ℃.

Sections underwent three rinses (2 min each) prior to overnight incubation in rabbit anti-c-Fos (catalogue #226 003, Synaptic Systems, Goettingen, Germany), diluted 1:1000 in PBS with 0.25% Triton-X (catalogue T9484, Sigma-Aldrich, St. Louis, MO, USA) and 0.01% sodium azide (D5637, Sigma-Aldrich, St. Louis, MO, USA). Following two 2 min rinses, sections incubated for 30 min in biotinylated donkey anti-rabbit secondary (code #711-065-152, Jackson Immunoresearch, West Grove, PA, USA) diluted 1:1000 in PBS. Sections underwent four additional rinses before incubating for 1 h in Vectastain ABC kit (Vector Laboratories, Burlingame, CA, USA) diluted 1:1000 in PBS. Sections rinsed twice before exposure to PBS containing 0.05% diaminobenzidine tetrahydrochloride hydrate (DAB; catalogue #D5637, Sigma-Aldrich, St. Louis, MO, USA), 0.05% ammonium nickel (II) sulfate hexahydrate (catalogue #A-1827, Sigma-Aldrich, St. Louis, MO, USA), and 0.01% hydrogen peroxide (30% in water) (catalogue #BP2633500, Fisher Scientific, Fair Lawn, NJ, USA). The stain was finalized by rinses in PBS.

### 2.3. C-Fos Quantification

Sections were imaged using a Nikon e400 biological microscope with a digital sight ds-u1 imaging attachment (Nikon Instruments Inc., Melville, NY, USA). Brain region coordinates were identified with use of The Mouse Brain in Stereotaxic Coordinates atlas [13]. Images were then analyzed using Icy bioimage informatics open source software version 1.0 (Institut Pasteur and France-BioImaging, Paris, France) [14]. Each c-Fos value represents 2–6 bilateral images per animal averaged together. Images were independently quantified by 2–3 investigators blind to genotype and drug treatment of each animal. The results generated by each investigator were then averaged together to produce the final value for each animal to insure lack of bias during image quantification.

### 2.4. Statistics

Analyses were performed in GraphPad Prism 8.0 (GraphPad Software, La Jolla, CA, USA). One-way analysis of variance (ANOVA) and Dunnett’s post hoc test were used to assess relative c-Fos reactivity across genotypes in saline treated animals. A two-way ANOVA (genotype × dose) and Tukey’s multiple comparisons test were used to assess relative c-Fos activation in each genotype following the 2 or 3 g/kg ethanol injection. Significance was defined as *p* < 0.05. Because of loss of tissue due to damage noted above, there was not sufficient sample size to detect sex effects. Given previous work on ethanol-induced CTA in these lines observed no sex-dependent differences and thus reported results of analyses collapsed on sex [4], we likewise collapsed data across sex in the present analyses.

## 3. Results

### 3.1. C-Fos Response in the Hindbrain

A2 numbers: HS saline, *n* = 8 (F = 4, M = 4), 2 g/kg, *n* = 10 (F = 5, M = 5), 3 g/kg, *n* = 8 (F = 5, M = 3); iHDID1 saline, *n* = 5 (F = 2, M = 3), 2 g/kg, *n* = 9 (F = 6, M = 3), 3 g/kg, *n* = 9 (F = 4, M = 5); iHDID2 saline, *n* = 9 (F = 5, M = 4), 2 g/kg, *n* = 9 (F = 4, M = 5), 3 g/kg, *n* = 9 (F = 4, M = 5). Locus coeruleus (LC) numbers: HS saline, *n* = 5 (F = 1, M = 4), 2 g/kg, *n* = 9 (F = 4, M = 5), 3 g/kg, *n* = 7 (F = 5, M = 2); iHDID1 saline, *n* = 7 (F = 3, M = 4), 2 g/kg, *n* = 9 (F = 5, M = 4), 3 g/kg, *n* = 8 (F = 3, M = 5); iHDID2 saline, *n* = 5 (F = 2, M = 3), 2 g/kg, *n* = 7 (F = 2, M = 5), 3 g/kg, *n* = 9 (F = 5, M = 4). Parabrachial nucleus (PBN) numbers: HS saline *n* = 9 (F = 4, M = 5), 2 g/kg, *n* = 8 (F = 4, M = 4), 3 g/kg, *n* = 5 (F = 3, M = 2); iHDID1 saline, *n* = 6 (F = 3, M = 3), 2 g/kg, *n* = 7 (F = 3, M = 4), 3 g/kg, *n* = 10 (F = 5, M = 5); iHDID2 saline, *n* = 7 (F = 3, M = 4), 2 g/kg, *n* = 8 (F = 3, M = 5), 3 g/kg, *n* = 11 (F = 6, M = 5).

**Figure 1:**
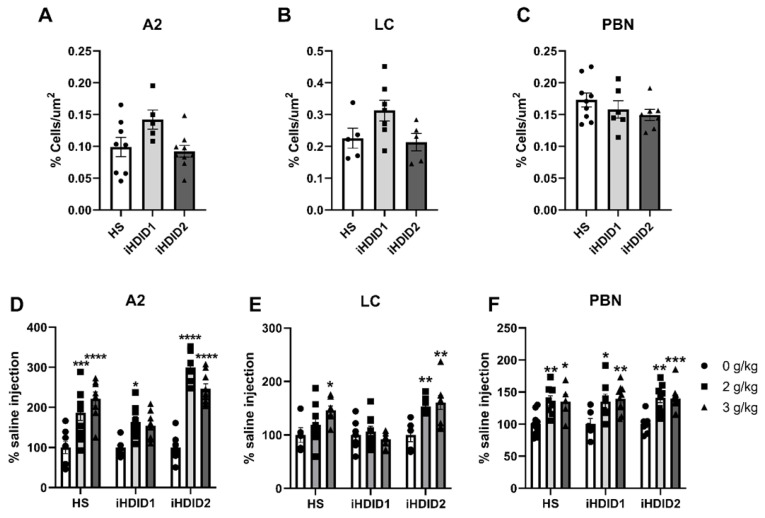
C-Fos Levels in the Hindbrain. (**A**–**C**) No difference in saline-induced c-Fos activation was detected between genotypes in any area. (**D**) Ethanol induced increased c-Fos levels in the A2 at all doses in the HS and iHDID2, but not at the 3 g/kg dose in the iHDID1 line. (**E**) Ethanol induced changes in c-Fos levels relative to saline in the HS and iHDID2, but not iHDID1 in the LC. (**F**) Ethanol induced increased c-Fos levels in the PBN in all genotypes. * *p* < 0.05, ** *p* < 0.01, *** *p* < 0.001, **** *p* < 0.0001 from two-way ANOVA Tukey’s multiple comparisons test.

C-Fos immunoreactivity following injection of 0 (saline) was assessed by one-way ANOVA and Dunnett’s post hoc test in male and female HS, iHDID1, and iHDID2 mice to assess potential baseline differences in regional activity in response to injection. One-way ANOVA detected no significant differences between genotypes in the A2 (F (2, 19) = 3.466, *p* = 0.0521), LC (F (2, 14) = 3.165, *p* = 0.0734), PBN (F (2, 19) = 1.248, *p* = 0.3097) Figure 1A–C.

**Figure 2:**
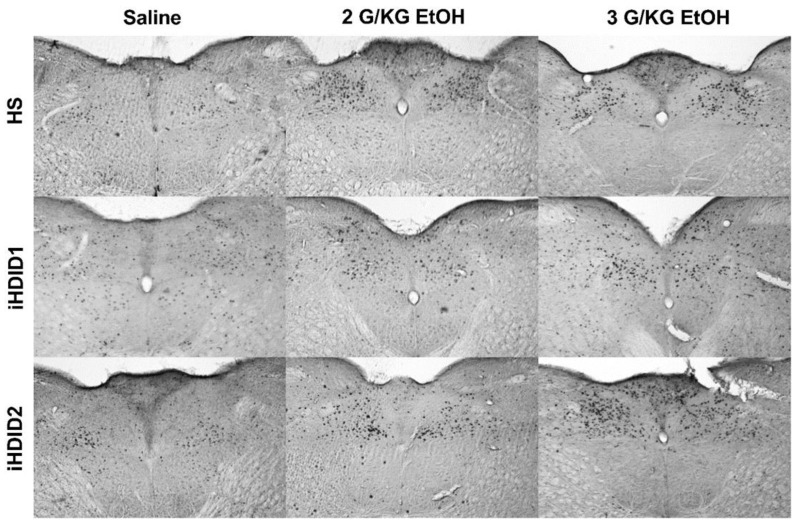
A2 Region of the Solitary Tract Nucleus. Representative images of c-Fos expression in the A2 across genotype and treatment types.

**Figure 3:**
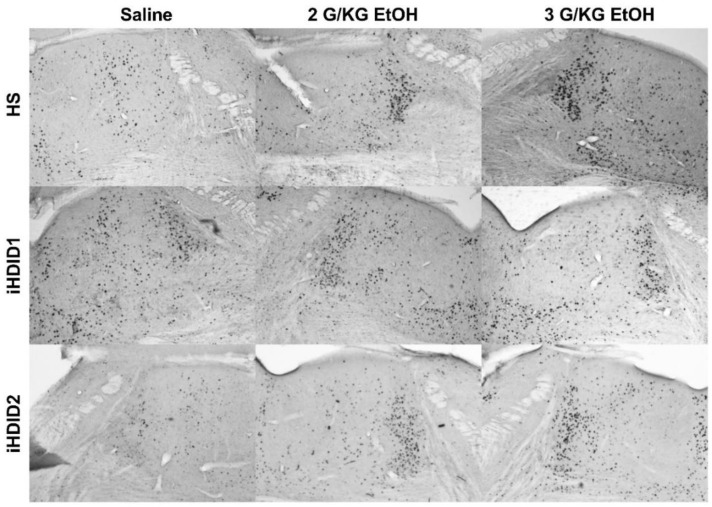
Locus Coeruleus. Representative images of c-Fos expression in the LC across genotype and treatment types.

**Figure 4:**
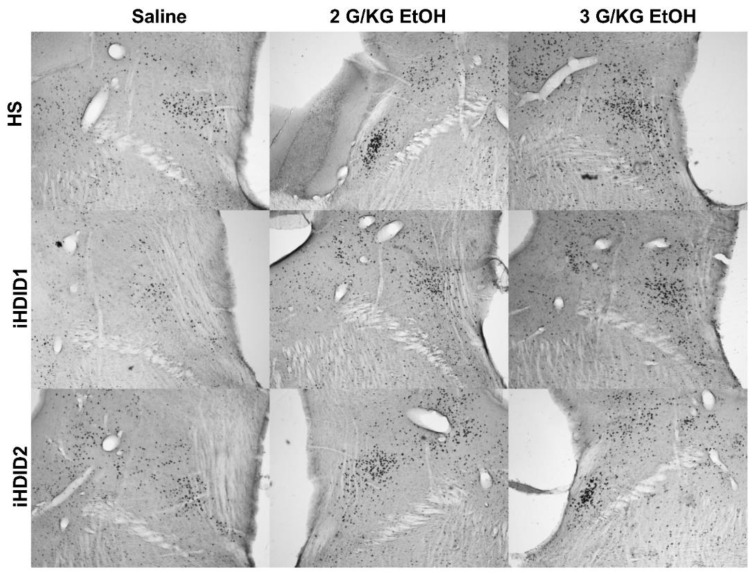
Parabrachial Nucleus. Representative images of c-Fos expression in the PBN across genotype and treatment types.

The change in percent c-Fos expression relative to the saline injection was then assessed following a 2 or 3 g/kg ethanol injection with two-way ANOVA (genotype × dose) and Tukey’s multiple comparisons test. In the A2, a significant effect of genotype (F (2, 67) = 19.96, *p* < 0.0001), dose (F (2, 67) = 53.47, *p* < 0.0001) and an interaction of genotype × dose (F (4, 67) = 7.093, *p* < 0.0001) were detected. Post hoc analysis found a significant increase in c-Fos expression relative to saline injection in the HS (2 g/kg ethanol: *p* = 0.0001; 3 g/kg ethanol: *p* < 0.0001), iHDID1 (2 g/kg ethanol: *p* = 0.0209; 3 g/kg ethanol: *p* = 0.0589), and iHDID2 (2 g/kg ethanol: *p* < 0.0001; 3 g/kg ethanol: *p* < 0.0001) genotypes (Figure 1D and Figure 2). In the LC, a significant impact of genotype (F (2, 57) = 9.648, *p* = 0.0002), dose (F (2, 57) = 6.596, *p* = 0.0027), and an interaction of genotype × dose (F (4, 57) = 3.137, *p* = 0.0212) were detected. Post hoc analysis found a significant increase in c-Fos expression relative to saline injection in the HS (2 g/kg ethanol: *p* = 0.4805; 3 g/kg ethanol: *p* = 0.0257) and iHDID2 (2 g/kg ethanol: *p* = 0.0076; 3 g/kg ethanol: *p* = 0.0013), but not in the iHDID1 (2 g/kg ethanol: *p* = 0.9044; 3 g/kg ethanol: *p* = 0.8550) genotypes (Figure 1E and Figure 3). In the PBN, a significant impact of dose (F (2, 62) = 22.12 *p* < 0.0001), but not genotype (F (2, 62) = 0.1425, *p* = 0.8675) nor an interaction (F (4, 62) = 0.08052, *p* = 0.9880) was detected. Post hoc analysis found a significant increase in c-Fos expression relative to saline injection in the HS (2 g/kg ethanol: *p* = 0.0030; 3 g/kg ethanol: *p* = 0.0159), iHDID1 (2 g/kg ethanol: *p* = 0.0135; 3 g/kg ethanol: *p* = 0.0022), and iHDID2 (2 g/kg ethanol: *p* = 0.0015; 3 g/kg ethanol: *p* = 0.0010) genotypes (Figure 1F and Figure 4).

### 3.2. C-Fos Response in the Extended Amygdala

Central amygdala (CeA) and basolateral amygdala (BLA) numbers: HS saline, *n* = 6 (F = 3, M = 3), 2 g/kg, *n* = 7 (F = 2, M = 5), 3 g/kg, *n* = 9 (F = 5, M = 4); iHDID1 saline, *n* = 7 (F = 3, M = 4), 2 g/kg, *n* = 10 (F = 5, M = 5), 3 g/kg, *n* = 8 (F = 4, M = 4); iHDID2 saline, *n* = 8 (F = 5, M = 3), 2 g/kg, *n* = 6 (F = 3, M = 3), 3 g/kg, *n* = 8 (F = 4, M = 4). bed nucleus of the stria terminalis (BNST) numbers: HS saline, *n* = 5 (F = 2, M = 3), 2 g/kg, *n* = 6 (F = 3, M = 3), 3 g/kg, *n* = 7 (F = 5, M = 2); iHDID1 saline, *n* = 5 (F = 3, M = 2), 2 g/kg, *n* = 7 (F = 3, M = 4), 3 g/kg, *n* = 5 (F = 2, M = 3); iHDID2 saline, *n* = 7 (F = 4, M = 3), 2 g/kg, *n* = 7 (F = 3, M = 4), 3 g/kg, *n* = 6 (F = 4, M = 2).

**Figure 5:**
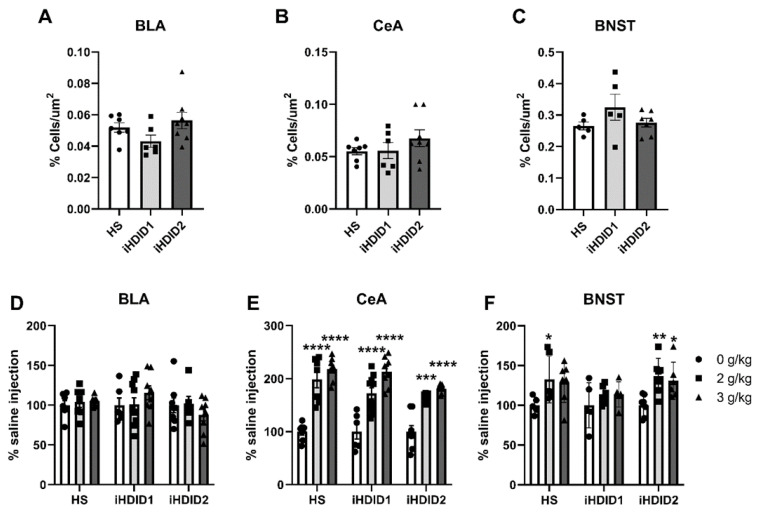
C-Fos Levels in the Extended Amygdala. (**A**–**C**) No difference in saline-induced c-Fos activation was detected between genotypes in any area. (**D**) Ethanol induced no change in c-Fos levels relative to saline in any genotype in the BLA. (**E**) Ethanol induced robust changes in c-Fos levels relative to saline in any genotype in the CeA. (**F**) Ethanol induced increased c-Fos levels in the BNST in the HS and iHDID2, but not iHDID1 line. * *p* < 0.05, ** *p* < 0.01, *** *p* < 0.001, and **** *p* < 0.0001 from two-way ANOVA Tukey’s multiple comparisons test.

C-Fos immunoreactivity following injection of 0 (saline) was assessed by one-way ANOVA and Dunnett’s post hoc test in male and female HS, iHDID1, and iHDID2 mice to assess potential baseline differences in regional activity in response to injection. One-way ANOVA detected no significant differences between genotypes in the BLA (F (2, 18) = 2.324, *p* = 0.1265), CeA (F (2, 18) = 1.146, *p* = 0.3399), or BNST (F (2, 14) = 1.614, *p* = 0.2341) (Figure 5A–C).

**Figure 6:**
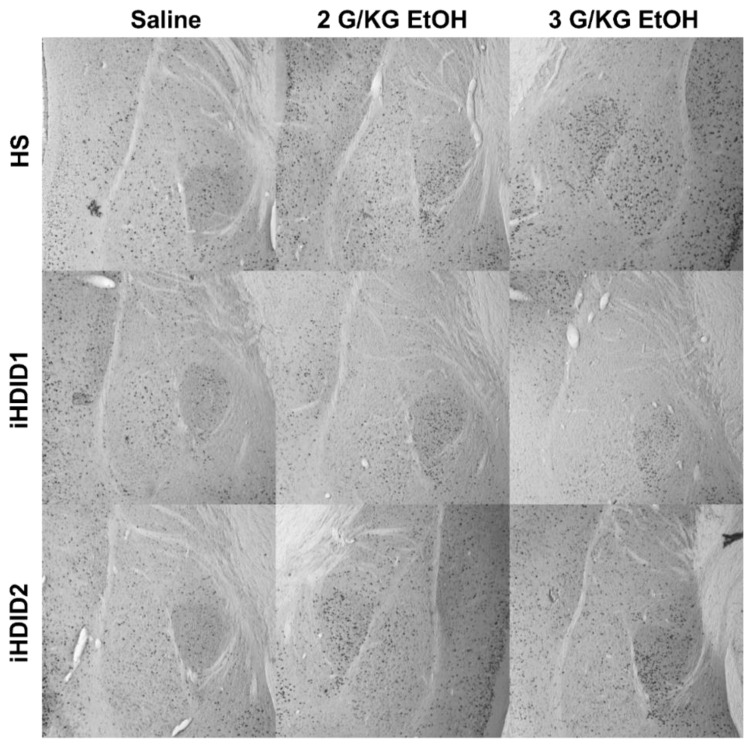
Basolateral and Central Amygdala. Representative images of c-Fos expression in the BLA and CeA across genotype and treatment types.

**Figure 7:**
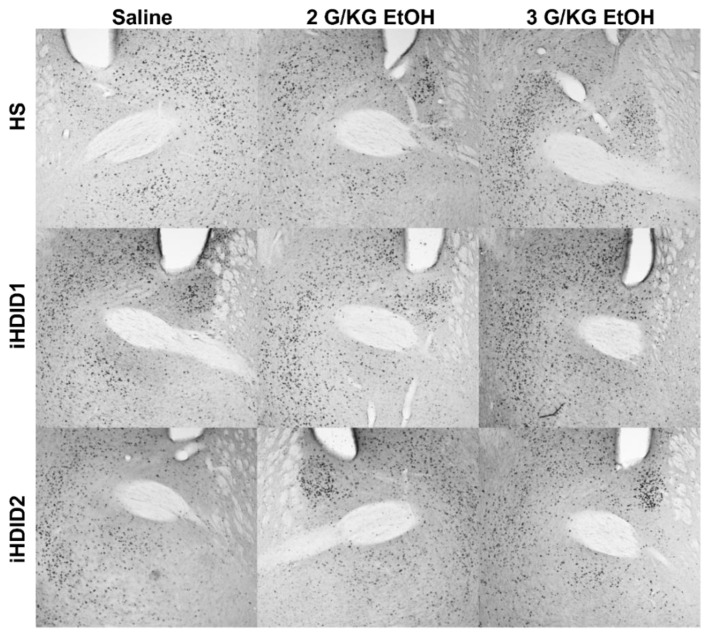
Bed Nucleus of the Stria Terminalis. Representative images of c-Fos expression in the BNST across genotype and treatment types.

The change in percent c-Fos expression relative to the saline injection was then assessed following a 2 or 3 g/kg ethanol injection with two-way ANOVA (genotype × dose) and Tukey’s multiple comparisons test. In the BLA, no significant impact of genotype (F (2, 59) = 1.011, *p* = 0.3700), dose (F (2, 59) = 0.1134, *p* = 0.8930), or interaction (F (4, 59) = 1.128, *p* = 0.3521) was detected (Figure 5D and Figure 6). In the CeA, a significant impact of genotype (F (2, 59) = 3.855, *p* = 0.0267) and dose (F (2, 59) = 84.06, *p* < 0.0001), but not an interaction (F (4, 59) = 1.487, *p* = 0.2176), were detected. Post hoc analysis found a significant increase in c-Fos expression relative to saline injection in the HS (2 g/kg ethanol: *p* < 0.0001; 3 g/kg ethanol: *p* < 0.0001), iHDID1 (2 g/kg ethanol: *p* < 0.0001; 3 g/kg ethanol: *p* < 0.0001), and iHDID2 (2 g/kg ethanol: *p* = 0.0001; 3 g/kg ethanol: *p* < 0.0001) genotypes (Figure 5E and Figure 6). In the BNST, a significant impact of dose (F (2, 46) = 8.907, *p* = 0.0005), but not genotype (F (2, 46) = 2.233, *p* = 0.1187) nor an interaction (F (4, 46) = 0.6160, *p* = 0.6533) was detected. Post hoc analysis found a significant increase in c-Fos expression relative to saline injection in the HS (2 g/kg ethanol: *p* = 0.0368; 3 g/kg ethanol: *p* = 0.0597) and iHDID2 (2 g/kg ethanol: *p* = 0.0052; 3 g/kg ethanol: *p* = 0.0282), but not in iHDID1 (2 g/kg ethanol: *p* = 0.5772; 3 g/kg ethanol: *p* = 0.5560) genotypes (Figure 5F and Figure 7).

### 3.3. C-Fos Response in the Thalamic Area

Lateral and medial habenula (LHb and MHb) numbers: HS saline, *n* = 8 (F = 5, M = 3), 2 g/kg, *n* = 6 (F = 3, M = 3), 3 g/kg, *n* = 7 (F = 4, M = 3); iHDID1 saline, *n* = 4 (F = 2, M = 2), 2 g/kg, *n* = 7 (F = 4, M = 3), 3 g/kg, *n* = 6 (F = 2, M = 4); iHDID2 saline, *n* = 6 (F = 3, M = 3), 2 g/kg, *n* = 3 (F = 2, M = 1), 3 g/kg, *n* = 7 (F = 4, M = 3). Paraventricular nucleus of the thalamus (PVT) numbers: HS saline, *n* = 7 (F = 4, M = 3), 2 g/kg, *n* = 6 (F = 3, M = 3), 3 g/kg, *n* = 7 (F = 5, M = 2); iHDID1 saline, *n* = 7 (F = 3, M = 4), 2 g/kg, *n* = 9 (F = 4, M = 5), 3 g/kg, *n* = 6 (F = 2, M = 4); iHDID2 saline, *n* = 7 (F = 3, M = 4), 2 g/kg, *n* = 4 (F = 2, M = 2), 3 g/kg, *n* = 8 (F = 4, M = 4).

**Figure 8:**
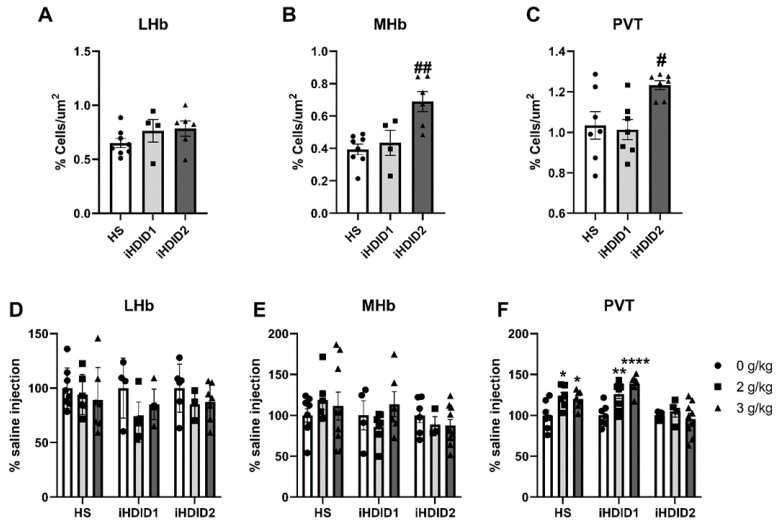
C-Fos Levels in the Midbrain. (**A**) No difference in saline-induced c-Fos activation was detected between genotypes in the LHb. (**B**,**C**) A significant increase in saline-induced c-Fos levels were detected in the iHDID2, but not iHDID1, line in the MHb and PVT relative to the HS genotype. (**D**,**E**) Ethanol induced no change in c-Fos levels relative to saline in any genotype in the LHb or MHb. (**F**) Ethanol induced increased c-Fos levels in the PVT in the HS and iHDID1, but not iHDID2 line. # *p* < 0.05, ## *p* < 0.01 from one-way ANOVA Dunnett’s multiple comparisons test; * *p* < 0.05, ** *p* < 0.01, and **** *p* < 0.0001 from two-way ANOVA Tukey’s multiple comparisons test.

C-Fos immunoreactivity following injection of 0 (saline) was assessed by one-way ANOVA and Dunnett’s post hoc test in male and female HS, iHDID1, and iHDID2 mice to assess potential baseline differences in regional activity in response to injection. One-way ANOVA detected no significant differences between genotypes in the LHb (F (2, 15) = 1.374, *p* = 0.2831). One-way ANOVA detected a significant difference between genotypes in the MHb (F (2, 15) = 9.650, *p* = 0.0020) in which post hoc revealed a significant difference between HS and iHDID2 (*p* = 0.0014), but not iHDID1 (*p* = 0.8408). One-way ANOVA detected a significant difference between genotypes in the PVT (F (2, 18) = 5.832, *p* = 0.0112) in which post hoc revealed a significant difference between HS and iHDID2 (*p* = 0.0220), but not iHDID1 (*p* = 0.9389) (Figure 8A–C).

**Figure 9:**
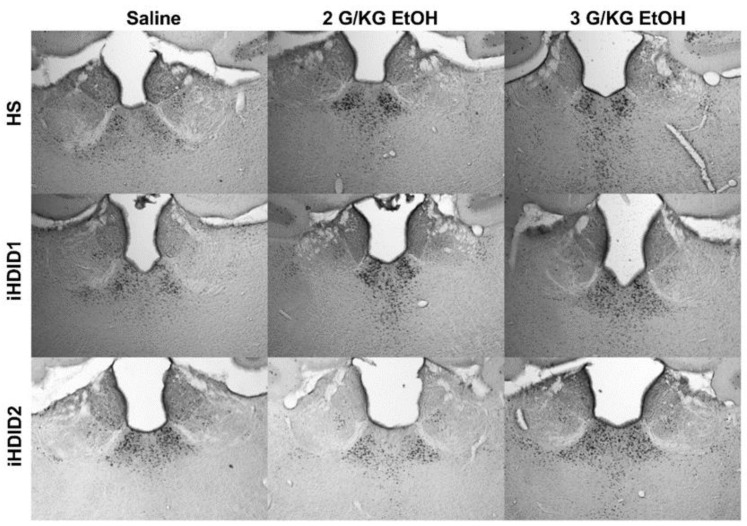
Lateral and Medial Habenula and Paraventricular Nucleus of the Thalamus. Representative images of c-Fos expression in the LHb, MHb, and PVT across genotype and treatment types.

The change in percent c-Fos expression relative to the saline injection was then assessed following a 2 or 3 g/kg ethanol injection with two-way ANOVA (genotype × dose) and Tukey’s multiple comparisons test. In the LHb, no significant impact of genotype (F (2, 45) = 1.002, *p* = 0.3754), dose (F (2, 45) = 2.975, *p* = 0.0612), nor an interaction (F (4, 45) = 0.6694, *p* = 0.6166) was detected (Figure 8D and Figure 9). In the MHb, no significant impact of genotype (F (2, 50) = 1.496, *p* = 0.2338), dose (F (2, 50) = 0.1997, *p* = 0.8196), nor an interaction (F (4, 50) = 1.015, *p* = 0.4089) was detected (Figure 8E and Figure 9). In the PVT, a significant effect of genotype (F (2, 53) = 10.67, *p* = 0.0001), dose (F (2, 53) = 10.41, *p* = 0.0002) and an interaction of genotype × se (F (4, 53) = 4.038, *p* = 0.0062) were detected. Post hoc analysis found a significant increase in c-Fos expression relative to saline injection in the HS (2 g/kg ethanol: *p* = 0.0193; 3 g/kg ethanol: *p* = 0.0358), iHDID1 (2 g/kg ethanol: *p* = 0.0024; 3 g/kg ethanol: *p* < 0.0001), but not iHDID2 (2 g/kg ethanol: *p* = 0.8514; 3 g/kg ethanol: *p* = 0.8304) genotypes (Figure 8F and Figure 9).

### 3.4. C-Fos Response in Other Regions Examined

Periaqueductal gray (PAG): HS saline, *n* = 7 (F = 4, M = 3), 2 g/kg, *n* = 5 (F = 3, M = 2), 3 g/kg, *n* = 8 (F = 4, M = 4); iHDID1 saline, *n* = 5 (F = 2, M = 3), 2 g/kg, *n* = 8 (F = 3, M = 5), 3 g/kg, *n* = 6 (F = 3, M = 3); iHDID2 saline, *n* = 7 (F = 3, M = 4), 2 g/kg, *n* = 6 (F = 2, M = 4), 3 g/kg, *n* = 10 (F = 5, M = 5). Edinger–Westphal nuclei (EW) numbers: HS saline, *n* = 6 (F = 4, M = 2), 2 g/kg, *n* = 4 (F = 3, M = 2), 3 g/kg, *n* = 9 (F = 5, M = 4); iHDID1 saline, *n* = 5 (F = 3, M = 2), 2 g/kg, *n* = 9 (F = 7, M = 2), 3 g/kg, *n* = 6 (F = 3, M = 3); iHDID2 saline, *n* = 6 (F = 2, M = 4), 2 g/kg, *n* = 6 (F = 3, M = 3), 3 g/kg, *n* = 10 (F = 5, M = 5). Rostromedial tegmental nucleus (RMTg) numbers: HS saline, *n* = 9 (F = 5, M = 4), 2 g/kg, *n* = 8 (F = 4, M = 4), 3 g/kg, *n* = 8 (F = 5, M = 3); iHDID1 saline, *n* = 7 (F = 3, M = 4), 2 g/kg, *n* = 10 (F = 5, M = 5), 3 g/kg, *n* = 6 (F = 2, M = 4); iHDID2 saline, *n* = 7 (F = 4, M = 3), 2 g/kg, *n* = 6 (F = 3, M = 3), 3 g/kg, *n* = 10 (F = 5, M = 5).

**Figure 10:**
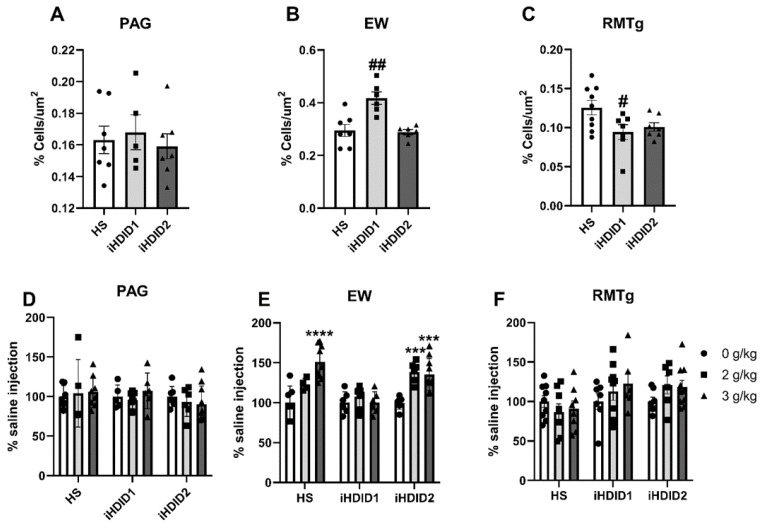
C-Fos Levels in Other Regions Examined. (**A**) No difference in saline-induced c-Fos activation was detected between genotypes in the PAG. (**B**,**C**) A significant difference in saline-induced c-Fos levels were detected in the iHDID1, but not iHDID2, line in the EW and RMTg relative to the HS genotype. (**D**) Ethanol induced no change in c-Fos levels relative to saline in any genotype in the PAG. (**E**) Ethanol induced changes in c-Fos levels relative to saline in the HS and iHDID2, but not iHDID1 in the EW. (**F**) Ethanol induced no change in c-Fos levels relative to saline in any genotype in the RMTg. # *p* < 0.05, ## *p* < 0.01 from one-way ANOVA Dunnett’s multiple comparisons test; *** *p* < 0.001, and **** *p* < 0.0001 from two-way ANOVA Tukey’s multiple comparisons test.

C-Fos immunoreactivity following injection of 0 (saline) was assessed by one-way ANOVA and Dunnett’s post hoc test in male and female HS, iHDID1, and iHDID2 mice to assess potential baseline differences in regional activity in response to injection. One-way ANOVA detected no significant differences between genotypes in the PAG (F (2, 16) = 0.2242, *p* = 0.8016). One-way ANOVA detected a significant difference between genotypes in the EW (F (2, 16) = 12.37, *p* = 0.0006) in which post hoc revealed a significant difference between HS and iHDID1 (*p* = 0.0011), but not iHDID2 (*p* = 0.9611). One-way ANOVA detected a significant difference between genotypes in the RMTg (F (2, 20) = 4.018, *p* = 0.0341) in which post hoc revealed a significant difference between HS and iHDID1 (*p* = 0.0296), but not iHDID2 (*p* = 0.0906) (Figure 10A–C).

**Figure 11:**
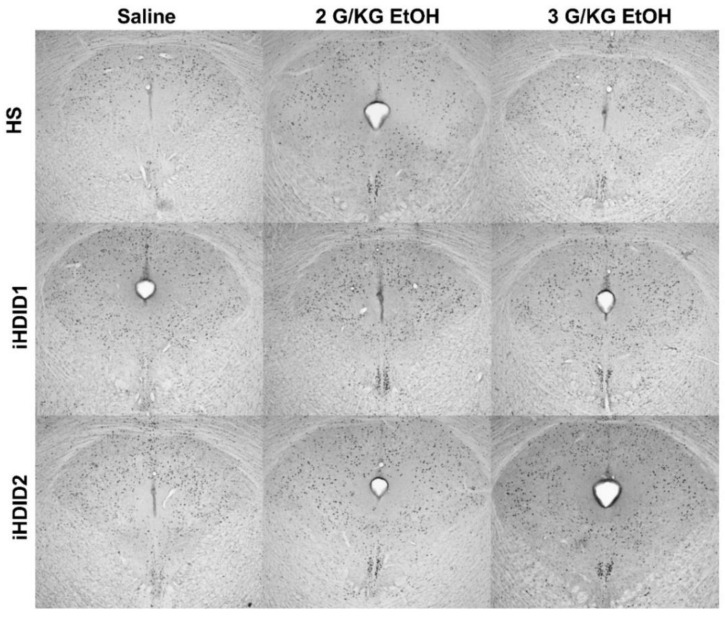
Periaqueductal Gray. Representative images of c-Fos expression in the PAG across genotype and treatment types.

**Figure 12:**
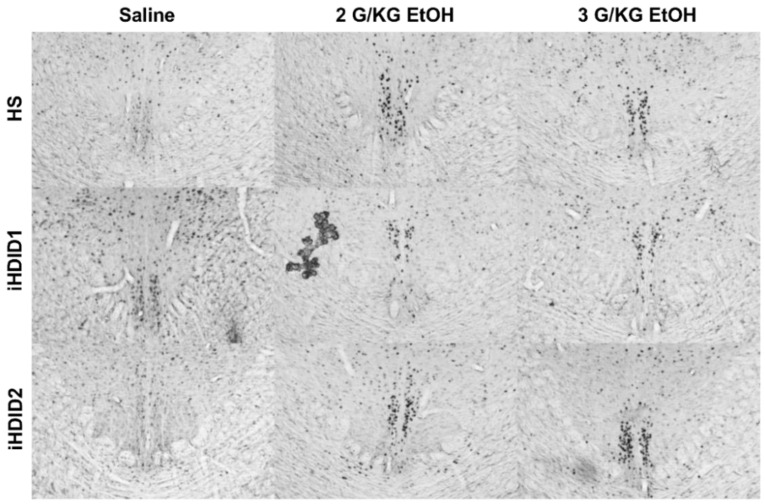
Edinger Westphal Nucleus. Representative images of c-Fos expression in the EW across genotype and treatment types.

**Figure 13:**
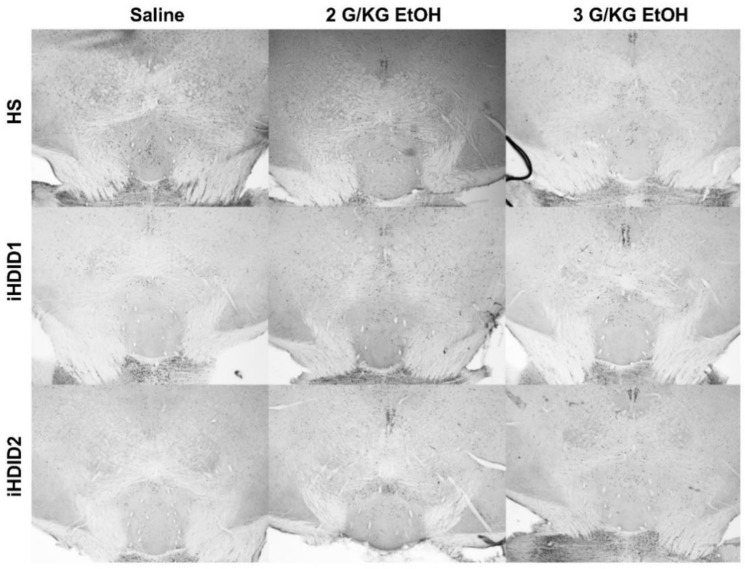
Rostromedial Tegmental Nucleus. Representative images of c-Fos expression in the RMTg across genotype and treatment types.

The change in percent c-Fos expression relative to the saline injection was then assessed following a 2 or 3 g/kg ethanol injection with two-way ANOVA (genotype × dose) and Tukey’s multiple comparisons test. In the PAG, no significant impact of genotype (F (2, 53) = 0.9948, *p* = 0.3766), dose (F (2, 53) = 0.1637, *p* = 0.8494), nor an interaction (F (4, 53) = 0.5041, *p* = 0.7329) was detected (Figure 10D and Figure 11). In the EW, a significant effect of genotype (F (2, 53) = 12.00, *p* < 0.0001), dose (F (2, 53) = 17.41, *p* < 0.0001), and an interaction of genotype × dose (F (4, 53) = 5.277, *p* = 0.0012) was detected. Post hoc analysis found a significant increase in c-Fos expression relative to saline injection in the HS (2 g/kg ethanol: *p* = 0.0740; 3 g/kg ethanol: *p* < 0.0001) and iHDID2 (2 g/kg ethanol: *p* = 0.0004; 3 g/kg ethanol: *p* = 0.0003), but not iHDID1 (2 g/kg ethanol: *p* = 0.6829; 3 g/kg ethanol: *p* > 0.9999) genotypes (Figure 10E and Figure 12). In the RMTg, a significant impact of genotype (F (2, 62) = 4.122, *p* = 0.0208) but not dose (F (2, 62) = 0.8829, *p* = 0.4187) nor an interaction (F (4, 62) = 1.095, *p* = 0.3668) was detected. Tukey’s multiple comparisons test detected no significant differences in any genotype (*p* > 0.05) (Figure 10F and Figure 13).

## 4. Discussion

In this work, we have demonstrated both overlapping and significantly divergent patterns of ethanol-induced c-Fos activation in the iHDID1 and iHDID2 lines of mice, relative to their HS parent strain (see Table 1 for summary of results). These findings complement previous work demonstrating an extensive difference in patterns of c-Fos activation in genetic lines bred for a high ethanol preference relative to low-preference lines [10,11,15]. Notably, c-Fos activation in select regions differed between the iHDID1 and iHDID2 lines, lending strength to the hypothesis that polygenetic factors underlie the same behavioral end point in these two replicate inbred lines, as previously observed in assessment of brain gene coexpression networks of the founding HDID1 and HDID2 strains [6]. Specifically, c-Fos activation in the iHDID1 line was found to differ from the HS line either at baseline or following ethanol exposure in the BNST, A2, LC, EW, and to a lesser extent the RMTg. In contrast, the HDID2 line differed compared to the HS line in the PVT and MHb. It is notable that while the iHDID1 and iHDID2 lines showed similar c-Fos activity as the HS line in multiple brain regions, in no region did the two both significantly differ from the HS line together. An important limitation to the present work is that there was not sufficient sample size to detect potential sex differences ethanol-induced effects on c-Fos expression, and it will be important to address this variable in future research. Below we provided additional consideration for differences in ethanol-induced c-Fos expression that we observed between these lines. It is of note the iHDID lines were bred specifically for binge-ethanol behavior, which cannot be generalized to other forms of intake. Indeed, the HDID1 line has been found to ingest lower amounts of high concentration ethanol relative to the HS founder line [16], highlighting that an increase in one form of ethanol intake in no way suggests an increase in all ethanol-directed behaviors. Given this caveat, we include literature related to genetic lines bred for overall higher ethanol intake/preference to offer additional context in discussion and to note brain regions involved in binge-ethanol intake may be critical across multiple stages/patterns of ethanol intoxication.

### 4.1. Hindbrain Regions

Previous work has demonstrated that iHDID lines exhibit decreased sensitivity to the aversive properties of ethanol when compared to the control lines, but do not show altered sensitivity to the rewarding properties of ethanol [10,11,15]. Based on these observations, the doses of ethanol used in the present work were specifically chosen to examine potential mechanisms underlying altered sensitivity to the aversive effects of ethanol. Specifically, we selected doses that have been shown to induced blunted ethanol-induced CTA in iHDID1 and iHDID2 relative to the parent HS line [4]. Hindbrain regions, included the nucleus of tract solitary (NTS) and LC, have been shown to be active in response to stimuli that can support CTA (for review see [17]). The involvement of the A2, a subregion of the NTS, in CTA is thought to be due to noradrenaline (NE) efferents to the PBN, a brain area that is strongly related to deleterious somatosensory and visceral insults [18] (see below). The A2 noradrenergic neurons further modulate seeking behaviors for drugs of abuse [19]. Previous work has found acute ethanol exposure induces A2 c-Fos expression in mice and rats [20,21,22]. Interestingly, while both ethanol doses induced increased c-Fos in the A2 in the HS and iHDID2 line, only the low dose of ethanol induced c-Fos activation in the iHDID1 line, suggesting that ethanol-induced activation of the A2 is blunted in iHDID1 mice. Given the role of the NTS in aversion learning, it is tempting to speculate that blunted ethanol-induced activation of the A2 in iHDID1 mice may contribute to their reduced sensitivity to the aversive effects of ethanol. However, since iHDID2 mice similarly show reduced sensitivity to the aversive effects of ethanol, a link between the A2 sensitivity to the aversive properties of ethanol is not straightforward. Regardless, blunted ethanol-induced c-Fos expression in the A2 of iHDID1 mice relative to the HS strain suggests a potential mechanism involved in high binge-like ethanol intake in the iHDID1 line.

The LC is a catecholamine nucleus and NE is its primary neurotransmitter [23]. With extensive projections throughout the central nervous system [24], this brainstem region has a major role in stress-related behaviors [25], and is involved in the shaping of alcohol-drinking behaviors [21,26]. It is known that aversive states induce LC activation, including ethanol in doses that support CTA [27,28,29,30]. Our findings show variations in the pattern of c-Fos expression on LC induced by acute ethanol, as the iHDID1 line mice displayed no LC activation, while iHDID2 animals presented LC activation after both doses of ethanol. Interestingly, variances in the sensitivity of LC to acute ethanol treatment have been observed between alcohol-preferring (P) and alcohol-non-preferring (NP) lines, and the Alko-alcohol (AA) and Alko-non-alcohol (ANA) lines, with high-drinking lines exhibiting blunted ethanol-induced c-Fos expression relative to low-drinking lines [31]. Given work linking the LC to the modulation of the aversive properties of ethanol, the reduced activation of the LC observed in the iHDID1 animals may contribute to their reduced sensitivity to the aversive properties of ethanol, and this blunted response may serve as a key mechanism driving binge-like ethanol consumption specifically in the iHDID1 line. However, similar to the A2, since iHDID2 mice also show reduced sensitivity to the aversive effects of ethanol, the link between the LC and sensitivity to the aversive properties of ethanol is not straightforward with respect to the iHDID lines.

Acute ethanol induced significant increases in PBN c-Fos expression across all lines. This finding is in keeping with numerous other studies in rodents utilizing a range of acute ethanol doses [22,32,33,34]. The precise role of the PBN in ethanol consumption is not fully understood. However, in addition to the role in CTA outlined above, recent work has demonstrated stimulation of a neurotensin expressing CeA-PBN pathway increases ethanol, but not water or quinine intake, and modulates the reinforcing properties of ethanol [35]. This pathway is likely intact in the iHDID1 and iHDID2 animals as, in addition to similar c-Fos expression in the PBN, there were also no difference in ethanol-induced c-Fos expression in the CeA between lines. Given the blunting of A2/LC activation observed in the iHDID1 line, this may lend further support to the hypothesis that pathways involved in processing the rewarding properties of ethanol (CeA-PBN) remain unaltered in the iHDID1 line while pathways mediating the aversive properties (A2/LC-PBN) are impaired.

### 4.2. Extended Amygdala Regions

Given the importance of the CeA in all stages of ethanol abuse and the general assignment of salience to both rewarding and aversive stimuli, it is of little surprise similar patterns of c-Fos activity were observed across all three lines [35,36,37]. Inhibitory GABAergic cells within the CeA are known to respond to acute ethanol exposure across multiple species and genetic lines, as has been extensively reviewed previously [37,38,39]. Our present findings add to this large body of literature demonstrating an important role of the CeA across the ethanol abuse spectrum.

Also well known to modulate ethanol-directed behaviors and the anxiolytic effects of ethanol, in contrast to the GABAergic CeA, the BLA is composed primarily of glutamatergic pyramidal neurons which are known to be inhibited, rather than activated, by acute ethanol exposure [40]. As these glutamatergic cells display low tonic baseline activation, a decrease in c-Fos levels may be difficult to detect [41]. Thus, the lack of change in c-Fos activity following ethanol exposure in all lines is somewhat expected.

The most notable finding in the areas of the extended amygdala analyzed is the significant increase in BNST c-Fos expression following ethanol exposure in the HS and iHDID2, but not the iHDID1 line. The BNST is known to play a role in modulating neurobiological responses to both appetitive and aversive stimuli [42]. The acute ethanol-induced increase in BNST c-Fos expression observed in the HS and iHDID2 lines is consistent with previous works in Long–Evans and Sprague–Dawley rats and C57BL6/J mice [21,43,44,45,46]. The precise impact of impaired ethanol-induced c-Fos expression in the BNST in the iHDID1 line on the drive to consume ethanol is presently unknown. Chronic stress exposure has been shown to attenuate acute ethanol-induced cAMP response element-binding protein (CREB) phosphorylation in the BNST [47]. Further, disruption of glutamatergic signaling within the BNST induces an insensitivity to the aversive properties of ethanol [48]. Together, these results indicate that during acute ethanol exposure, such as used in this work, a primary role of BNST activation may be the processing of the stress-inducing, or aversive, properties of high dose ethanol. The BNST receives numerous glutamatergic and GABAergic afferents, with the CeA known to exert significant control over BNST stress-related signaling (for review see [42,49]). As the CeA response to acute ethanol was not different between lines in this study, the lack of BNST response in the iHDID1 line is likely due to alterations in the BNST itself rather than alterations in CeA signaling to the BNST. This could be due to alterations in signaling from other afferent areas, or changes in receptor expression within the BNST which receive incoming CeA transmission. The CeA is known to modulate BNST activity via both GABAergic and numerous neuropeptide signals, with corticotrophin releasing factor (CRF) and Neuropeptide Y (NPY) being perhaps the most well studied regarding ethanol use disorder research [37,38,50]. It would be of future interest to analyze potential alterations in BNST NPY/CRF/GABA receptor expression levels in the iHDID1 line relative to the HS and iHDID2 lines. Notably, NPY expression in the CeA and BNST display significant baseline or ethanol-induced alterations in expression in the HDID lines relative to the HS line, highlighting a potential critical role of this neuropeptide in driving binge-like ethanol consumption in these lines [51]. In contrast, it is important to note lesioning of the BNST or selective pharmacological silencing of BNST projections to the VTA decreases binge-like ethanol intake [52,53,54]. The precise mechanisms underlying the genetic predisposition towards impaired BNST activation following acute ethanol exposure require further investigation.

### 4.3. Midbrain Regions

No baseline differences or ethanol-induced changes in c-Fos were detected in the LHb. Similar to the above regions, the LHb has been shown to be critically involved in ethanol-induced CTA, with CTA expression promoting an elevation in tonic LHb firing and lesion of the LHb sufficient to block CTA development [55]. Indeed, the LHb plays an overall important role in processing of aversive stimuli and negative reinforcement (for review see [56]). LHb lesion further increases voluntary ethanol consumption and operant responding [57]. Consistent with this, LHb electric stimulation has been found to reduce voluntary ethanol consumption [58]. Interestingly, a lack of change in LHb c-Fos expression following ethanol consumption was found in the Li and colleagues work, with potent alterations detected only during withdrawal from ethanol exposure [58]. Our findings of no change in LHb c-Fos expression between the lines following acute ethanol exposure are in keeping with this previous work. Thus, it would be of future interest to observe if LHb c-Fos expression in the iHDID lines is blunted relative to the HS line during withdrawal from ethanol. Interestingly, in Sprague–Dawley rats, low dose acute ethanol injection (0.25 g/kg) significantly increased LHb c-Fos in glutamatergic cells, suggesting a potentially dose-dependent role of this area in response to acute exposure which may also be of future interest in evaluation of the iHDID lines [59].

No ethanol-induced changes in MHb c-Fos were detected in any of the genetic lines; however, the iHDID2 line was found to express significantly higher levels of c-Fos following a saline injection relative to the HS strain. The MHb has been recently put forward as a critical area in modulating drug abuse behavior (for review see [60]). The MHb serves to modulate reward information as a relay station in the transmission of signals from the limbic forebrain to the midbrain and hindbrain [61]. Interestingly, baseline increased glucose utilization rate and a total lack of the inhibitory peptide NPY are found in the MHb of ethanol-preferring relative to non-preferring rodent genetic lines [62,63]. Further, the NPY system is one of the genes found to show significant changes in connectivity in HDID mice relative to the founder HS stock, and further HDID1 and HDID2 mice display distinct baseline, as well as blunted ethanol-induced alterations in, NPY expression in a region-specific manner [6,51]. This may suggest that MHb hyperactivity contributes to ethanol-directed behaviors across multiple genetic lines. The precise impact of this hyperactivity has yet to be investigated. Previous work has found mice will voluntarily stimulate the MHb, and that ablation of this region reduces sucrose preference [64]. Interestingly, the MHb metabolic activity was found to be significantly increased in rats following exposure to chronic unpredictable mild stress and for MHb lesion to in fact increase sucrose preference in these animals [65]. This suggests either a difference in MHb role between species, or a potentially state dependent role of the MHb in mediating reward/anhedonia associated behaviors. This present work offers the first evidence that MHb baseline activity may contribute to increased ethanol consumption in the iHDID2, but not iHDID1, lines of mice.

The RMTg is another region closely associated with aversive signaling. While no ethanol-induced alterations in RMTg c-Fos were observed in any line, an interesting significant reduction in RMTg activity following saline injection alone was observed in the iHDID1 line relative to the parent HS line. The RMTg serves to regulate ventral tegmental area (VTA) activity via extensive GABAergic innervation (for review see [66]). Critically, this region is thought to participate in limiting voluntary ethanol intake through potent regulation of the aversive properties of ethanol. For example, RMTg lesion results in increased voluntary ethanol consumption and accelerates CTA extinction [67]. Though initially somewhat surprising, the lack of ethanol-induced alteration in c-Fos detected in this work is consistent with previous findings in which only ethanol paired with a CTA environment, but not ethanol administered without this pairing, induced an increase in RMTg c-Fos [68]. It is likely that assessment following voluntary ethanol consumption within an ethanol-paired environment, in contrast to ethanol administered by IP injection in this work, would offer more relevant insight into potential differences in ethanol-induced alterations in RMTg c-Fos expression between the HS and iHDID lines. Interestingly, recent work has suggested that the densely expressed Mu-opioid receptor (MOR) population in the RMTg may play a central role in regulating the balance between the aversive/rewarding properties of ethanol [69,70]. This is notable as HDID1 ethanol consumption has been shown to be insensitive to naltrexone (a MOR-antagonist) administration [71]. Taken together, the reduced RMTg activity at baseline detected in this work may suggest that altered MOR regulation of activity in this area serves as a brain region contributing to this naltrexone insensitivity.

### 4.4. Other Regions Examined

PAG activity has previously been shown to be modulated by ethanol exposure. For instance, acute ethanol increases glutamatergic transmission onto PAG dopaminergic neurons in ex vivo recordings, and binge-like ethanol exposure in alcohol-preferring rats alters PAG RNA expression [72,73]. Given the role of the PAG in processing pain, anxiety, and reward processing, and the apparent attenuation of the aversive effects of ethanol in the iHDID lines, we sought to assess any alteration in PAG reactivity to a saline injection or following ethanol exposure relative to the HS line (for review see [74]). No differences between lines following saline or ethanol exposure were detected, suggesting a potential lack of PAG involvement in processing of an acute ethanol injection in all lines. This finding is in keeping with previous work which found no change in PAG c-Fos expression following lower dose ethanol exposure in rats [45]. However, given the highly heterogenous composition of the region, it is possible only a subset of PAG cells are engaged by acute ethanol exposure and thus may not be detected by the general assessment of total c-Fos activity. Future work will be required to parse out the specific role of the PAG in the regulation of binge drinking behavior.

The EW likely plays a critical role in modulating ethanol-directed behaviors, as ethanol, but not water, saccharine, or non-alcoholic beer, intake induces increased activity in this region [21,75]. We found acute ethanol exposure increased EW c-Fos expression in the HS and iHDID2 lines, but not the iHDID1 line. Of note, the iHDID1 line expressed significantly higher levels of EW c-Fos expression following a saline injection alone relative to the HS line. This increased activity is unlikely to be due to an increased sensitivity of the iHDID1 line to the stressful stimuli of an IP saline injection, as previous work has shown stress exposure does not induce an increase in EW c-Fos expression [76]. This lack of an ethanol-induced increase in c-Fos expression is therefore potentially due to a ceiling effect of high basal EW activity in the iHDID1 line rather than an insensitivity to ethanol. This region expresses numerous neuromodulators which may contribute to its role in binge-like drinking behavior (for review see [77]. The dense levels of urocortin (Ucn1), a neuropeptide which binds to CRF receptors, expression is the most extensively studied in relation to drug abuse behavior [78]. Indeed, previous studies have found that ethanol induced c-Fos expression in the EW to predominantly colocalize with Ucn1 expression in C57BL/6J mice [79]. Increased levels of Ucn1 expression have been found in numerous rodent lines known for high ethanol preference and intake (for review see [78]). Though Ucn1 levels have not been directly assessed in iHDID1 animals, an increased baseline level of Ucn1 as found in other genetic lines would be in keeping with the higher baseline levels of EW c-Fos activity in the iHDID1 line.

Of regions assessed in this work, the PVT stands as the lone area in which the iHDID2 line differed in ethanol-induced c-Fos expression from the HS founder line. The PVT has been put forward as an integration hub for drug-related hypothalamic signaling [80,81,82]. The PVT is a primarily glutamatergic structure which sends projections to brain regions known to be critical to ethanol-related behavior, such as the extended amygdala, nucleus accumbens, and prefrontal cortex [82]. Distinct inputs to the PVT have been shown to play a role in reward-related behaviors. Specifically, the orexin system potently regulates PVT signaling and is suggested to primarily act in this region in regulating negative emotional states (for review see [80]; prefrontal cortex glutamatergic inputs have been shown to modulate cue-reward learning [83]; while stimulation of zona incerta GABAergic inputs to the PVT rapidly stimulates binge-food consumption [84]. This role in reward learning/seeking behavior may be of particular interest in ethanol-directed behaviors. Lesion of or kappa-opioid receptor agonism into the PVT prevents reinstatement of ethanol seeking behavior, with this behavior potentially regulated by PVT projections to the nucleus accumbens [85,86,87]. Interestingly, the PVT is suggested to be involved in the encoding, and not retrieval, of emotionally salient information [85,88]. This is of particular note as in this work the lack of ethanol-induced increase in PVT c-Fos in the iHDID2 line is likely due to the significantly higher baseline activation of the PVT observed in this line following saline injection. This suggests that c-Fos levels could not be increased following ethanol exposure due to a ceiling effect. This general heightened activity of the PVT may thus result in a non-discriminatory assignment of salience to stimuli and increased drive to seek consumable substances. While only speculation, such an interpretation would be in keeping with the previously observed non-specific increase in consumption of ethanol, water, and saccharine, as well as the specific increase in bout number, but not bout duration, observed in HDID2 mice [15]. Future studies examining the precise role of the PVT in driving the seeking and consumption of ethanol and other substances in the iHDID2 line would be of particular interest.

## 5. Conclusions

The results of this work contribute, in combination with previous studies, to characterizing the potential neural mechanisms underlying the drive to binge consume ethanol in two replicate iHDID lines. Our findings lend further support to the previously proposed hypothesis the iHDID1 and the iHDID2 lines arrive at a similar behavior phenotype through divergent genetic mechanisms [6]. Assessing genetic mechanisms underlying resistance to the aversive effects of ethanol may be of particular clinical relevance as sensitivity to aversive ethanol effects such as dizziness and nausea is less severe in sons of alcoholics relative to sons of non-alcoholics, with this constitutional difference proposed to be a factor influencing the difference in risk for alcoholism in these two groups [89]. Given the increasing importance of personalized medicine in treating alcohol use disorder in humans, the iHDID replicate lines stand as an important model in evaluating both potential pharmacotherapies and of the multiple potential mechanisms underlying ethanol-directed behaviors between individuals.

## Figures and Tables

**Table 1:** Summary of significant differences in vehicle- or ethanol-induced c-Fos expression between genotypes. Arrow indicates increase (↑) or decrease (↓) in c-Fos expression. A2: A2 region of the nucleus of tract solitary (NTS); LC: locus coeruleus; PBN: parabrachial nucleus; BLA: basolateral amygdala; CeA: central amygdala; BNST: bed nucleus of the stria terminals; LHb: lateral habenula; MHb: medial habenula; PVT: paraventricular nucleus of the thalamus; PAG: periaqueductal gray; EW: Edinger–Westphal nuclei; RMTg: rostromedial tegmental nucleus * *p* < 0.05, ** *p* < 0.01, *** *p* < 0.001, and **** *p* < 0.0001 (see results for statistical analysis performed).

Region	Genotype	VEH c-Fos vs. HS VEH	2 G/KG EtOH c-Fos vs. VEH	3 G/KG EtOH c-Fos vs. VEH
**A2**	HS		↑ ***	↑ ****
	iHDID1	-	↑ *	-
	iHDID2	-	↑ ****	↑ ****
**LC**	HS		-	↑ *
	iHDID1	-	-	-
	iHDID2	-	↑ **	↑ **
**PBN**	HS		↑ **	↑ *
	iHDID1	-	↑ *	↑ **
	iHDID2	-	↑ **	↑ ***
**BLA**	HS		-	-
	iHDID1	-	-	-
	iHDID2	-	-	-
**CeA**	HS		↑ ****	↑ ****
	iHDID1	-	↑ ****	↑ ****
	iHDID2	-	↑ ***	↑ ****
**BNST**	HS		↑ *	-
	iHDID1	-	-	-
	iHDID2	-	↑ **	↑ *
**LHb**	HS		-	-
	iHDID1	-	-	-
	iHDID2	-	-	-
**MHb**	HS		-	-
	iHDID1	-	-	-
	iHDID2	↑ **	-	-
**PVT**	HS		↑ *	↑ *
	iHDID1	-	↑ **	↑ ****
	iHDID2	↑ *	-	-
**PAG**	HS		-	-
	iHDID1	-	-	-
	iHDID2	-	-	-
**EW**	HS		-	↑ ****
	iHDID1	↑ **	-	-
	iHDID2	-	↑ ***	↑ ***
**RMTg**	HS		-	-
	iHDID1	↓ *	-	-
	iHDID2	-	-	-
